# Preoperative Leg Muscle Quality Association Functional Recovery After Adult Spinal Deformity Surgery: A Propensity-Score-Matched Study

**DOI:** 10.3390/medicina61060980

**Published:** 2025-05-26

**Authors:** Tomoyoshi Sakaguchi, Masato Tanaka, Shinya Arataki, Tadashi Komatsubara, Akiyoshi Miyamoto, Aditya Thakur, Muhamad Aulia Rahman, Masato Tanaka, Kazuhiko Takamatsu, Yosuke Yasuda, Hidemi Fuji, Eri Oikawa, Moeka Ueda

**Affiliations:** 1 Department of Rehabilitation, Okayama Rosai Hospital, 1-10-25 Chikkō Midorimachi, Minami-ku, Okayama 702-8055, Japan; tomoyoshi0127@gmail.com (T.S.); kazuhikopt0803@gmail.com (K.T.); kyushudanji19861007@gmail.com (Y.Y.); hidemint.f@gmail.com (H.F.); yunax1214x@gmail.com (E.O.); mo6583260@gmail.com (M.U.); 2 Department of Orthopaedic Surgery, Okayama Rosai Hospital, Okayama 702-8055, Japan; araoyc@gmail.com (S.A.); t.komatsubara1982@gmail.com (T.K.); hello.akkun.11.136@icloud.com (A.M.); dr.adityathakur@gmail.com (A.T.); auliarahmanmd@gmail.com (M.A.R.); 3 Department of Radiology, Okayama Rosai Hospital, 1-10-25 Chikkō Midorimachi, Minami-ku, Okayama 702-8055, Japan; tanaka0896@gmail.com

**Keywords:** adult spinal deformity, timed up and go test, muscle quality, functional cross-sectional area

## Abstract

*Background and Objectives:* We conducted a retrospective observational study. *Background:* While traditional rehabilitation approaches emphasize trunk muscle conditioning, emerging evidence suggests that leg muscle strength plays a critical role in postoperative functional mobility. Previous studies have focused on trunk muscle rehabilitation in patients with adult spinal deformity (ASD). However, recent findings suggest that leg muscle conditioning may be a better predictor of postoperative improvement. Strengthening the gluteal and iliopsoas muscles has been linked to improved sagittal balance, gait stability, and mobility, ultimately leading to enhanced surgical outcomes. This study examines the relationship between the preoperative functional cross-sectional area (FCSA) of trunk and leg muscles and postoperative improvement in mobility, as assessed by the Timed Up and Go (TUG) test, in patients undergoing surgery for ASD. *Materials and Methods:* Sixty-two patients (57 women, mean age 71.2 ± 7.1 years) who underwent ASD surgery between April 2017 and April 2024 were retrospectively analyzed. The FCSA of key muscles—psoas major (PM), erector spinae (ES), multifidus (MF), gluteus maximus (GM), and gluteus medius (GMed)—was measured using CT images. Patients were categorized into an improvement group and a non-improvement group based on whether they achieved the minimum clinically important difference (MCID) of −2 s in the TUG test 12 months after surgery. Propensity score matching (PSM) was applied to adjust for baseline differences between the groups. A significance level of 5% was used for all comparisons. *Results:* Thirty-three patients achieved a clinically meaningful improvement in TUG, while 29 did not. Before PSM, patients with worse preoperative TUG and Oswestry Disability Index (ODI) scores showed greater improvements (*p* < 0.01). After PSM, no significant differences were found between the groups in terms of age, sex, or BMI. However, the improvement group showed significantly greater FCSA values in PM (6.1 ± 2.3 mm^2^ vs. 3.9 ± 1.5 mm^2^, *p* = 0.021) and GM (19.9 ± 5.9 mm^2^ vs. 15.3 ± 3.9 mm^2^, *p* = 0.019). *Conclusions:* This study demonstrates that leg muscle quality, particularly that of the gluteus maximus and psoas major, is a significant predictor of postoperative mobility improvement in patients with ASD. These findings challenge the conventional focus on trunk muscles and suggest the inclusion of leg muscle training in preoperative rehabilitation strategies to enhance surgical outcomes.

## 1. Introduction

With the increase in the aging population, the demand for the treatment of adult spinal deformity (ASD) has been growing [1]. The prevalence of ASD is reported to be as high as 68% among individuals aged 60 years and older [2]. The main symptoms of ASD include severe low back pain, impaired gait, and reduced balance, which significantly impact activities of daily living (ADL) and quality of life (QoL) [3]. ASD represents a complex set of spinal structural abnormalities affecting postural stability, sagittal balance, and overall mobility [4]. Muscle exercise therapy targeting the multifidus and erector spinae muscles is commonly used in the conservative management of ASD [5]. However, these muscles often undergo fatty degeneration after long spinal fusion [6], limiting their contribution to spinal balance. Furthermore, the surgery is also a fixation procedure that extends from the thoracic to the pelvic region, supporting the trunk muscles. Achieving optimal postoperative outcomes requires not only successful surgical correction, but also a well-defined rehabilitation strategy. Therefore, in patients awaiting long spinal fusion, preoperative interventions targeting alternative muscle groups may be warranted to enhance postoperative recovery. ASD surgery is highly invasive and often requires prolonged recovery [7]. Patients with ASD also exhibit impaired balance and a higher fall risk compared to other spinal disorders [8]. The accurate preoperative assessment of muscle function and early intervention may improve long-term outcomes.

Recent studies have highlighted that muscle quality and contractile capacity, rather than mass alone, are key predictors of surgical outcomes for patients with cancer or osteoporosis [9,10]. These can be quantified as the functional cross-sectional area (FCSA) using computed tomography (CT) or magnetic resonance imaging, excluding fatty infiltration [11], which provides a reliable measure of muscle function [11]. In ASD, FCSA has been linked to outcomes such as SRS-22 score improvement, infection, proximal junctional kyphosis, and rod breakage [12,13], but its relationship with functional recovery remains unclear. In other orthopedic fields, FCSA is associated with postoperative physical performance [14], suggesting a potential role in ASD as well.

The Timed Up and Go test (TUG) is a widely used functional assessment that reflects mobility and fall risk in ASD, and it correlates with postoperative Oswestry Disability Index (ODI) (Appendix A) more strongly than spinopelvic parameters [3,15]. Therefore, identifying patients with a low preoperative FCSA of trunk and leg muscles helps predict poor postoperative TUG improvement and allows for individualized preoperative planning. The patients who underwent long spinal fusion cannot use their trunk muscles effectively, so the authors set up a hypothesis that preoperative leg muscle quality is an important factor for patients’ clinical recovery after ASD surgery. By evaluating the FCSA of trunk and leg muscles relative to TUG improvements, we seek to provide clinically relevant insights into optimizing rehabilitation protocols.

## 2. Materials and Methods

**Figure 1:**
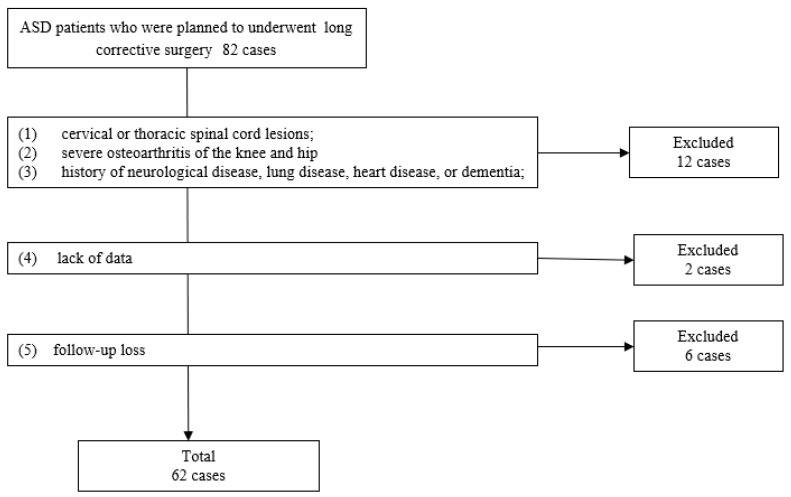
Patient selection.

This is a single-center retrospective study. The ethics committee of Okayama Rosai Hospital, Okayama, Japan, approved this study (approval no. 527-3; date of approval: 10 March 2025). Patients were informed of the study’s content, and their written consent was obtained. In total, 82 consecutive patients who underwent surgery for ASD at Okayama Rosai Hospital from April 2017 to April 2024 were included in this study. The inclusion criteria were (1) age ≥ 60 years; (2) patients who underwent corrective surgery from the thoracic spine to the pelvis; (3) patients who can walk more than 10 m; and (4) patients for whom informed consent was available. The exclusion criteria were (1) cervical or thoracic spinal cord lesions; (2) severe osteoarthritis of the knee and hip; (3) a history of neurological disease, lung disease, heart disease, or dementia; (4) a lack of data; and (5) follow-up loss. A total of 62 patients were included in this study (Figure 1).

Postoperative rehabilitation was conducted as follows. After the first-stage surgery, patients underwent bed-based physical therapy to prevent disuse syndrome while preparing for the second-stage surgery. The interval between the two surgical stages was one week.

Following the second-stage spinal fusion, patients began mobilization depending on pain levels, with a spinal brace (corset) in place. Physical therapy sessions lasting 40 min per day were continued until discharge. The physical therapists provided aerobic exercise, isometric strengthening exercises for the trunk and lower limbs, and instructions for activities of daily living. Patients were specifically instructed to avoid trunk flexion and rotation during daily movements.

TUG, ODI, and VAS for low back, buttock, and lower extremity pain were evaluated the day before the first-stage surgery and at the 12-month follow-up. Qualified physiotherapists assessed TUG. For radiological assessment, CT scans and radiographs obtained within one month before surgery were assessed by radiological staff.

### 2.1. Patient and Surgical Factors

Patient-related factors included sex, age, and body mass index (BMI). Surgical factors included surgical time, intraoperative blood loss, surgical type, and the upper instrumented vertebra (UIV). All patients underwent the same postoperative rehabilitation protocol.

### 2.2. Timed up and Go Test (TUG)

TUG was used to assess functional mobility [16]. Participants began seated in a standard armchair. Upon the verbal command “Go”, they stood up from the chair, walked a distance of 3 m at a comfortable pace, turned around at a marked point, walked back to the chair, and sat down. The time taken to complete this sequence was recorded in seconds. All patients were demonstrated the TUG procedure by qualified physical therapists. TUG was assessed preoperatively and 12 months after ASD surgery. The minimal clinically important difference (MCID) of TUG 12 months after ASD surgery is −2 s [17].

### 2.3. Functional Cross-Sectional Area (FCSA)

**Figure 2:**
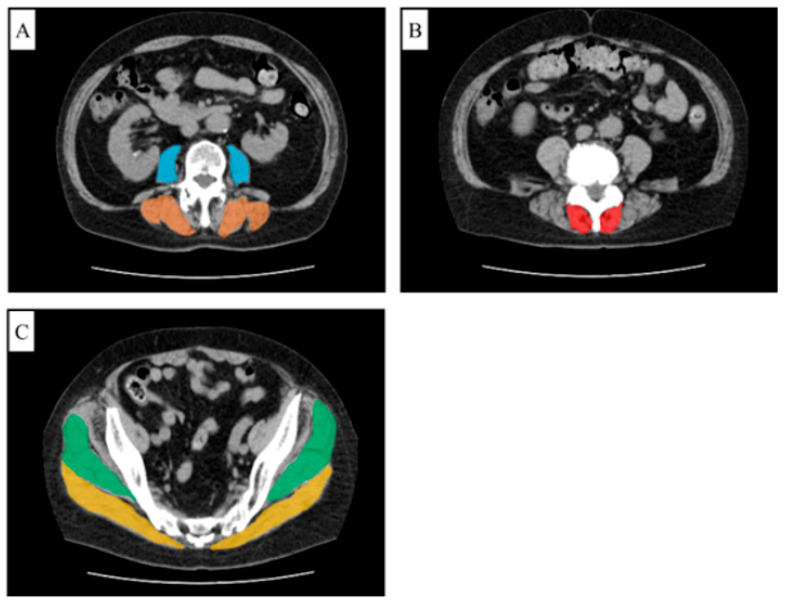
Measurement method of functional cross-sectional area of muscles. (**A**) Cross-sectional area of the psoas major (blue) and erector spinae (orange) at L3 level. (**B**) Cross-sectional area of the multifidus (red) at L4 level. (**C**) Cross-sectional area of the gluteus maximus (yellow) and gluteus medius (green) at S2 level.

CT images of the lumbar spine and lower limbs were obtained in the supine position with the knees slightly flexed using two different CT scanners, Aquilion PRIME and Aquilion Lightning (Toshiba Medical Systems, Otawara, Tochigi, Japan), before surgery. The images were acquired under a soft tissue window with a slice thickness of 5 mm. Using these images, the psoas major (PM), multifidus (MF), erector spinae (ES), gluteus maximus (GM), and gluteus medius (GMed) muscles were traced using image analysis software SYNAPSE VINCENT Ver. 7.0 (Fujifilm, Tokyo, Japan). The FCSA of each muscle was then calculated using a thresholding process based on Hounsfield unit (HU) values. The FCSA was measured in the following slices: the PM and ES at the L3 vertebral level, the MF at the L4 level, and the GM and GMed at the S2 level (Figure 2). The previously validated HU range for skeletal muscle (−29 to +150 HU) was applied to define the lean muscle area [18].

### 2.4. Patient-Reported Outcome

The Oswestry Disability Index (ODI) is a widely used questionnaire that assesses the degree of disability related to low back pain and serves as a reliable measure of health-related quality of life in patients with ASD [19]. The ODI consists of 10 items that evaluate different aspects of daily function, including pain intensity, personal care, lifting, walking, sitting, standing, sleeping, sexual activity, social life, and traveling. Each item is scored from 0 to 5, and the total score is converted into a percentage, with higher percentages indicating a greater degree of disability.

Pain levels in the low back, buttocks, and lower extremities were assessed using the Visual Analog Scale (VAS), a validated method to measure subjective pain intensity. Patients were asked to rate their pain on a scale of 0 (no pain) to 10 (the worst imaginable pain).

### 2.5. Spinopelvic Parameters

**Figure 3:**
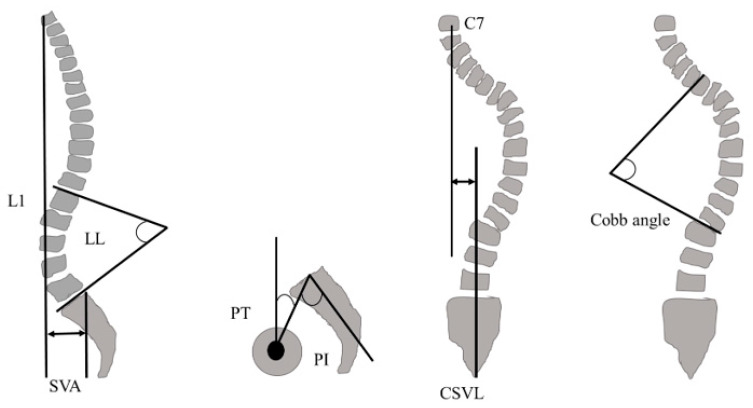
Spinopelvic parameters. SVA: sagittal vertical axis, LL: lumbar lordosis, PT: pelvic tilt, PI: pelvic incidence, CSVL: central sacral vertical line.

Spinopelvic parameters, including the sagittal vertical axis (SVA), lumbar lordosis (LL), pelvic tilt (PT), pelvic incidence (PI), Cobb angle, and central sacral vertical line (CSVL), were evaluated using radiographic imaging (Figure 3). Spinopelvic parameters were measured by a lateral spinal radiograph with the patient standing, hands placed on their clavicles (or chest), and the pelvis in a neutral position.

### 2.6. Statistical Analysis

Patients were classified into an improvement group and a non-improvement group based on a minimal clinically important difference (MCID) of 2 s in the TUG at 12 months postoperatively. Between-group comparisons of patients and surgical factors, as well as preoperative TUG, FCSA, ODI, VAS, and spinopelvic parameters, were performed using the Mann–Whitney U and chi-square tests. To adjust for baseline severity, propensity score matching was performed using age, sex, BMI, preoperative TUG, and ODI as covariates. After matching, between-group comparisons were conducted again. Data were processed using EZR version 1.61 [20]. After performing propensity score matching, a power analysis was conducted using G*Power to evaluate the statistical power of the group comparisons [21]. This analysis was performed to ensure the adequacy of the sample size and the ability to detect significant effects in the matched groups. A *p*-value of <0.05 was considered statistically significant.

## 3. Results

At 12 months postoperatively, we had 62 patients (57 female and 5 male) averaging 71.2 ± 7.1 years in our study. Based on the TUG outcome, 33 patients were classified into the improvement group and 29 into the non-improvement group. Before propensity score matching, the improvement group had significantly worse preoperative TUG and ODI scores than the non-improvement group (*p* < 0.01) (Table 1). After 1:1 propensity score matching, 13 patients were selected for each group. Following matching, the groups had no significant differences in preoperative TUG and ODI scores, indicating balanced baseline severity.

Post-matching comparisons revealed no significant differences in age, sex, and BMI. The average surgical time for IG and NIG was 477.9 min and 473.8 min, respectively. The average intraoperative blood loss for IG and NIG was 1380 mL and 1269 mL, respectively. There is a significant difference between the two groups during the first stage of surgery (*p* < 0.01) and in the preoperative FCSA of the PM and GM (*p* < 0.05) (Table 2).

A post hoc power analysis was performed for the three variables that showed statistically significant differences between the matched groups: estimated blood loss during the first surgery and the preoperative FCSA of GM and PM. Using G*Power (version 3.1), the achieved power (1-β) was calculated based on the observed effect sizes, with α = 0.05 and a sample size of 13 participants per group. The statistical power was sufficient for estimated blood loss (0.95), PM (0.87), and GM (0.74). However, the power was low for ES (0.51), MF (0.56), and GMed (0.16).

## 4. Discussion

In conservative treatment for adult spinal deformity (ASD), physical therapy focuses on the core and the back group of muscles [22]. However, this group of back muscles is prone to postoperative fatty degeneration due to surgical intervention, which may limit their ability to contribute to maintaining gait and balance [5,6]. Previous studies have shown that leg muscle strength is associated with postural sway during gait in patients after ASD surgery [23]. Therefore, the preoperative strengthening of the leg muscles might be necessary to enhance postoperative gait and balance performance in patients awaiting ASD surgery. Moreover, recent evidence suggests that lower limb muscle strength is more strongly associated with postoperative functional outcomes than trunk muscle strength in ASD patients [24]. However, most previous studies have not evaluated individual lower limb muscles separately, and it remains unclear whether specific muscles are associated with achieving the MCID in physical function after ASD surgery. Identifying which muscles contribute to MCID achievement could help inform targeted preoperative strengthening interventions. In this study, we used propensity score matching to minimize the influence of baseline severity and evaluated the FCSA of trunk and lower limb muscles in relation to MCID achievement in TUG after ASD surgery.

In the pre-matching analysis, the improved group, which achieved the MCID in TUG, showed significantly worse preoperative TUG and ODI scores than the non-improved group. This finding suggests that patients with greater functional impairment before surgery may exhibit more pronounced improvement postoperatively. To control for baseline severity, we performed propensity score matching using age, sex, BMI, preoperative TUG, and ODI as covariates and compared 13 cases in each group. After matching, no significant differences were observed between the two groups in preoperative TUG and ODI scores, allowing for comparisons under balanced baseline conditions. As a result, the improved group tended to have larger preoperative FCSA values for the PM and GM. Although the sample size after propensity score matching was relatively small (n = 13 per group), a post hoc power analysis revealed that the statistical power was acceptable for the key outcome variables that showed significant between-group differences. The power was 0.87 for the FCSA of the PM and 0.74 for the FCSA of the GM. These results support the robustness of the observed associations, particularly for the leg muscle parameters.

Recent studies have increasingly highlighted the importance of lower limb muscles in postural compensation and functional recovery in patients with ASD. Yamato et al. reported that compensatory mechanisms, such as pelvic retroversion and knee flexion during standing, are associated with increased muscle activity not only in the trunk, but also in the pelvis and lower limbs [25]. Furthermore, Banno et al. investigated muscle activity during gait before and after surgery in ASD patients and demonstrated a postoperative decrease in trunk muscle activation alongside an increase in lower limb muscle activity [26]. This shift reflects a greater reliance on lower limb muscles than trunk muscles for dynamic balance after surgery. These findings support our results, which showed that the preoperative FCSA of lower limb muscles was associated with postoperative improvement in the TUG. Given its functional role, the PM and GM may play a key role in postoperative mobility improvements.

The PM is a primary hip flexor and contributes to spinal stability and the maintenance of postural balance during leg movements [27,28]. Its dysfunction has been associated with poor outcomes on the ODI following lumbar spine surgery [29]. Also, its dysfunction increased SVA and gait instability in patients with ASD [23,30]. As spinal motion is often restricted after ASD surgery, the PM may compensate for impaired lumbar mobility by contributing to trunk control during gait [31]. Therefore, a larger preoperative FCSA of the PM may facilitate better postoperative mobilization, leading to improvements in TUG. This was evident in our study, as the patients in the improved group had an FCSA of PM 6.1 ± 2.3 cm^2^, whereas the non-improved group had an FCSA of PM 3.9 ± 1.5 cm^2^. This difference was statistically significant (*p* = 0.021).

Similarly, the GM also plays an essential role in maintaining an upright posture and facilitating bipedal locomotion [32]. Prior studies have shown that poor GM function is associated with the deterioration of spinopelvic alignment [33], while the postoperative strengthening of the GM may help prevent such deterioration [34]. In addition, GM function has been correlated with postoperative TUG performance in patients undergoing hip arthroplasty [35], further highlighting its contribution to dynamic balance. After spinal surgery, the multifidus and erector spinae muscles often atrophy, making the GM particularly important in compensating for postural control. In this context, a larger preoperative FCSA of the GM may contribute to maintaining upright posture after surgery. In our study, we also observed a significant statistical difference in the FCSA of the GM between the improved (FCSA of GM 19.9 ± 5.9 cm^2^) and non-improved groups (FCSA of GM 15.3 ± 3.9 cm^2^).

GMed is one of the important pelvic stabilizers, preventing it from dropping on the non-weight-bearing side during the swing phase of walking. The role of GMed in postural stabilization is to assist in upright trunk positioning, particularly for patients with weakened paraspinal musculature [30]. Lee et al. also reported the importance of strengthening the gluteal maximus muscle and increasing muscle volume after ASD, and the role of GMed is limited to providing balance aid as it does not significantly affect sagittal alignment [25]. These findings are consistent with our study; the FCSA of the GMed was not statistically different between the improved and non-improved groups.

Taken together, these findings suggest that the preoperative condition of trunk and hip stabilizing muscles, such as the PM and GM, may significantly influence functional recovery following ASD surgery. Preserving or enhancing these muscle groups could be a key strategy for improving dynamic balance in postoperative rehabilitation. Future studies should comprehensively evaluate the impact of surgical invasiveness, the degree of correction, and muscle mass on functional outcomes.

This study has several limitations. First, the small sample size after propensity score matching (n = 13 per group) may limit the statistical power to detect differences and reduce the generalizability of the findings. Second, this was a retrospective single-center study, which may introduce selection bias and limit external validity. Third, although the cross-sectional area of several muscles was evaluated, variability in the CT acquisition timing and muscle measurement levels may have affected the consistency of FCSA assessment. Fourth, only TUG was used to assess functional recovery without incorporating other necessary physical performance measures such as gait speed, balance, or ADL. Additionally, other potentially relevant lower limb muscles, such as the quadriceps, hamstrings, and triceps surae, were not assessed using radiological methods.

Future studies should involve prospective multicenter designs with larger sample sizes to enhance validity and generalizability. Randomized controlled trials should also be conducted to investigate whether preoperative lower extremity muscle strengthening interventions improve postoperative mobility outcomes. Furthermore, future research should explore the relationship between FCSA and various functional parameters, including walking speed, balance ability, and independence in daily activities.

## 5. Conclusions

This study demonstrated that the greater preoperative FCSA of the PM and GM was significantly associated with clinically meaningful improvement in mobility, as measured by the TUG test, 12 months after ASD surgery. These findings highlight the critical role of leg muscle quality, particularly the hip stabilizers, in predicting postoperative functional recovery. The assessment of FCSA in these muscles may serve as a valuable tool in preoperative risk stratification. Patients with low FCSA may benefit from targeted pre-rehabilitation focused on strengthening leg muscles to optimize surgical outcomes and enhance long-term mobility.

## Figures and Tables

**Table 1:** Comparison between improved and non-improved groups before propensity score matching.

Mean ± SD; *n*				
	Total (*n* = 62)	Improved Group (*n* = 33)	Non-Improved Group (*n* = 29)	*p* Value
Age (year)	71.2 ± 7.1	72.3 ± 6.5	70.0 ± 7.5	0.247
Sex				0.884
Men	5	2	3	
Women	57	31	26	
BMI	23.1 ± 3.6	22.9 ± 3.2	23.3 ± 3.9	0.914
Operation time (min)				
1st stage (OLIF)	196.4 ± 45.7	195.6 ± 47.5	197.3 ± 42.3	0.418
2nd stage (PCF)	283.6 ± 53.3	279.1 ± 50.4	288.6 ± 55.0	0.358
Bleeding (mL)				
1st stage (OLIF)	404.9 ± 306.9	444.2 ± 336.4	356.5 ± 251.1	0.395
2nd stage (PCF)	890.5 ± 539.7	738.1 ± 358.1	1046.7 ± 640.7	0.083
Type of posterior Fusion				0.209
OPEN	32	15	17	
MIS	30	18	12	
UIV				0.637
Th1	1	0	1	
Th4	1	0	1	
Th6	3	1	2	
Th9	1	1	0	
Th10	56	31	25	
TUG (sec)	12.6 ± 4.7	15.0 ± 4.9	9.71 ± 2.1	<0.01
VAS of LBP (mm)	56.8 ± 26.8	63.2 ± 22.2	49.2 ± 29.2	0.133
VAS of leg pain (mm)	25.7 ± 35.9	29.8 ± 35.4	19.3 ± 33.9	0.375
ODI (%)	40.3 ± 12.9	44.1 ± 12.8	36.1 ± 11.3	<0.01
FCSA				
PM (cm^2^)	5.0 ± 2.1	5.6 ± 2.2	4.5 ± 1.9	0.057
ES (cm^2^)	4.7 ± 12.7	7.4 ± 2.9	6.9 ± 2.6	0.781
MF (cm^2^)	3.7 ± 1.4	3.9 ± 1.3	3.5 ± 1.5	0.128
GM (cm^2^)	17.2 ± 5.8	17.1 ± 5.4	17.4 ± 6.2	0.861
GMed (cm^2^)	22.1 ± 4.2	22.2 ± 4.2	22.1 ± 4.3	0.345
Spinopelvic parameters				
SVA (mm)	118.2 ± 53.8	119.1 ± 49.9	117.1 ± 57.1	0.878
LL (degree)	11.4 ± 14.5	14.9 ± 16.1	7.3 ± 10.6	0.264
PT (degree)	35.8 ± 10.1	34.9 ± 10.1	36.9 ± 9.9	0.183
PI (degree)	52.7 ± 6.8	51.8 ± 7.1	53.5 ± 6.2	0.453
PI-LL (degree)	40.8 ± 15.6	36.7 ± 16.9	45.5 ± 11.9	0.195
Cobb (degree)	28.3 ± 21.9	28.2 ± 18.8	28.4 ± 24.5	0.233
CSVL (mm)	28.9 ± 24.3	30.9 ± 22.7	26.4 ± 25.3	0.348

BMI, body mass index; OLIF, oblique lateral interbody fusion; PCF, posterior corrective fusion; MIS, minimally invasive surgery; UIV, upper instrumented vertebra; TUG, Timed Up and Go test; LBP, low back pain; ODI, Oswestry Disability Index; FCSA, functional cross-sectional area; PM, psoas major; ES, erector spinae; MF, multifidus; GM, gluteus maximus; GMed, gluteus medius; SVA, sagittal vertical axis; LL, lumbar lordosis; PT, pelvic tilt; PI, pelvic incidence; Cobb, Cobb angle; CSVL, central sacral vertical line.

**Table 2:** Comparison between improved and non-improved groups after propensity score matching.

Mean ± SD; *n*				
	Total (*n* = 26)	Improved Group (*n* = 13)	Non-Improved Group (*n* = 19)	*p* Value
Age (year)	70.8 ± 6.3	70.6 ± 4.9	71.1 ± 7.4	0.797
Sex				1
Men	2	1	1	
Women	24	12	12	
BMI	23.2 ± 2.9	23.3 ± 2.4	23.0 ± 3.4	0.521
Operation time (min)				
1st stage (OLIF)	192.4 ± 45.3	194.3 ± 46.1	190.1 ± 44.4	0.849
2nd stage (PCF)	283.7 ± 59.4	283.6 ± 66.7	283.7 ± 51.1	0.358
Bleeding (mL)				
1st stage (OLIF)	364.8 ± 249.9	497.6 ± 252.8	220.8 ± 145.0	<0.01
2nd stage (PCF)	965.0 ± 605.6	881.9 ± 360.5	1048.1 ± 767.9	0.770
Type of posterior Fusion				1
OPEN	14	7	7	
MIS	12	6	6	
UIV				1
Th9	2	1	1	
Th10	24	12	12	
TUG (sec)	11.4 ± 1.5	11.3 ± 1.4	11.4 ± 1.7	0.921
VAS of LBP (mm)	56.0 ± 20.8	53.9 ± 20.1	59.1 ± 21.5	0.599
VAS of leg pain (mm)	20.6 ± 34.6	22.5 ± 38.9	18.7 ± 29.6	0.869
ODI (%)	42.6 ± 8.1	41.3 ± 7.0	43.9 ± 8.8	0.404
FCSA				
PM (cm^2^)	4.9 ± 2.2	6.1 ± 2.3	3.9 ± 1.5	0.021
ES (cm^2^)	6.8 ± 2.3	6.0 ± 2.5	7.5 ± 1.9	0.106
MF (cm^2^)	3.2 ± 1.1	3.5 ± 9.6	2.9 ± 1.2	0.119
GM (cm^2^)	17.5 ± 5.5	19.9 ± 5.9	15.3 ± 3.2	0.019
GMed (cm^2^)	21.9 ± 4.8	22.8 ± 4.2	21.5 ± 5.5	0.347
Spinopelvic parameters				
SVA (mm)	124.8 ± 56.3	115.1 ± 49.1	135.4 ± 63.2	0.663
LL (degree)	12.4 ± 10.3	14.6 ± 12.1	9.8 ± 6.9	0.341
PT (degree)	32.8 ± 8.6	34.0 ± 6.8	31.6 ± 9.9	0.877
PI (degree)	52.3 ± 6.3	50.5 ± 7.5	54.4 ± 5.3	0.199
PI-LL (degree)	39.8 ± 11.7	35.6 ± 12.8	44.4 ± 8.2	0.127
Cobb (degree)	52.8 ± 19.6	27.9 ± 20.1	23.5 ± 18.7	0.624
CSVL (mm)	24.9 ± 20.8	25.9 ± 16.2	23.8 ± 24.9	0.244

BMI, body mass index; OLIF, oblique lateral interbody fusion; PCF, posterior corrective fusion; MIS, minimally invasive surgery; UIV, upper instrumented vertebra; TUG, Timed Up and Go test; LBP, low back pain; ODI, Oswestry Disability Index; FCSA, functional cross-sectional area; PM, psoas major; ES, erector spinae; MF, multifidus; GM, gluteus maximus; GMed, gluteus medius; SVA, sagittal vertical axis; LL, lumbar lordosis; PT, pelvic tilt; PI, pelvic incidence; Cobb, Cobb angle; CSVL, central sacral vertical line.

## Data Availability

The original contributions presented in this study are included in the article. Further inquiries can be directed to the corresponding author.

## Abbreviations

**Abbreviation**	**Full Term**
ASD	Adult Spinal Deformity
ADL	Activities of Daily Living
QOL	Quality Of Life
FCSA	Functional Cross-Sectional Area
CT	Computed Tomography
TUG	Timed Up and Go Test
BMI	Body Mass Index
UIV	Upper Instrumented Vertebra
MCID	Minimal Clinically Important Difference
PM	Psoas Major
MF	Multifidus
ES	Erector Spinae
GM	Gluteus Maximus
GMed	Gluteus Medius
HU	Hounsfield unit
ODI	Oswestry Disability Index
VAS	Visual Analog Scale
SVA	Sagittal Vertical Axis
LL	Lumbar Lordosis
PT	Pelvic Tilt
PI	Pelvic Incidence
CSVL	Central Sacral Vertical Line
OLIF	Oblique Lateral Interbody Fusion
PCF	Posterior Corrective Fusion
MIS	Minimally Invasive Surgery
LBP	Low Back Pain

## Appendix A

**Oswestry Disability Index**

**Section 1—Pain intensity**	**Section 2—Personal care (washing, dressing etc)**
□ **I have no pain at the moment.** □ **The pain is very mild at the moment.** □ **The pain is moderate at the moment.** □ **The pain is fairly severe at the moment.** □ **The pain is very severe at the moment.** □ **The pain is the worst imaginable at the moment.**	□ **I can look after myself normally without causing extra pain.** □ **I can look after myself normally but it is very painful.** □ **It is painful to look after myself and I am slow and careful.** □ **I need some help but manage most of my personal care.** □ **I need help every day in most aspects of self care.** □ **I do not get dressed, wash with difficulty and stay in bed.**
**Section 3—Lifting**	**Section 4—Walking**
□ **I can lift heavy weights without extra pain.** □ **I can lift heavy weights but it gives extra pain.** □ **Pain prevents me from lifting heavy weights off the floor but I can manage if they are conveniently positioned, e.g. on a table.** □ **Pain prevents me from lifting heavy weights but I can manage light to medium weights if they are conveniently positioned.** □ **I can lift only very light weights.** □ **I cannot lift or carry anything at all.**	□ **Pain does not prevent me walking any distance.** □ **Pain prevents me walking more than 1 mile.** □ **Pain prevents me walking more than than 1/2 of a mile.** □ **Pain prevents me walking more than 100 yards.** □ **I can only walk using a stick or crutches.** □ **I am in bed most of the time and have to crawl to the toilet.**
**Section 5—Siting**	**Section 6—Standing**
□ **I can lift heavy weights without extra pain** □ **I can lift heavy weights but it gives extra pain** □ **Pain prevents me from lifting heavy weights off the floor, but I can manage if they are conveniently placed eg. on a table** □ **Pain prevents me from lifting heavy weights, but I can manage light to medium weights if they are conveniently positioned** □ **I can lift very light weights** □ **I cannot lift or carry anything at all**	□ **I can stand as long as I want without extra pain.** □ **I can stand as long as I want but it gives me extra pain.** □ **Pain prevents me from standing for more than 1 hour.** □ **Pain prevents me from standing for more than 1/2 an hour.** □ **Pain prevents me from standing for more than 10 minutes.** □ **Pain prevents me from standing at all.**
**Section 7—Sleeping**	**Section 8—Sex life (if applicable)**
□ **My sleep is never disturbed by pain.** □ **My sleep is occasionally disturbed by pain.** □ **Because of pain I have less than 6 hours sleep.** □ **Because of pain I have less than 4 hours sleep.** □ **Because of pain I have less than 2 hours sleep.** □ **Pain prevents me from sleeping at all.**	□ **My sex life is normal and causes no extra pain.** □ **My sex life is normal but causes some extra pain.** □ **My sex life is nearly normal but is very painful.** □ **My sex life is severely restricted by pain.** □ **My sex life is nearly absent because of pain.** □ **Pain prevents any sex life at all.**
**Section 9—Social life**	**Section 10—Travelling**
□ **My social life is normal and causes me no extra pain.** □ **My social life is normal but increases the degree of pain.** □ **Pain has no significant effect on my social life apart from limiting my more energetic interests, e.g. sport, etc.** □ **Pain has restricted my social life and I do not go out as often.** □ **Pain has restricted social life to my home.** □ **I have no social life because of pain.**	□ **I can travel anywhere without pain.** □ **I can travel anywhere but it gives extra pain.** □ **Pain is bad but I manage journeys over two hours.** □ **Pain restricts me to journeys of less than one hour.** □ **Pain restricts me to short necessary journeys under 30 minutes.** □ **Pain prevents me from travelling except to receive treatment.**

**Visual analog scale**
